# Why Neoliberalism Doesn't Spell the Death of Society: Commonality, Regulation, and the Politics of Social Cohesion

**DOI:** 10.1111/1468-4446.70031

**Published:** 2025-09-23

**Authors:** Jan Dobbernack

**Affiliations:** ^1^ Newcastle University Newcastle Upon Tyne UK

**Keywords:** governmentality, neoliberalism, social cohesion, social theory, Wendy Brown, ‘the social’

## Abstract

Perspectives on neoliberal political‐economic practice often frame its dominance in terms of harms to ‘society’. Prominently, Wendy Brown (2019, 52) offers an account of the ‘neoliberal revolution’, claiming that, when ‘the social vanishes from our ideas, speech, and experience’, commonality disappears, democracy diminishes, and authoritarianism prevails. The paper considers this understanding to argue for the importance of political articulations of ‘society’, which reveal complexities that elude nostalgic accounts of how the social has been lost. Making this case, it works through real‐world invocations of social commonality in the name of social cohesion. Social cohesion illustrates the multiplicity of objectives invoking ‘society’, ranging from the production of pro‐social subjects to the pursuit of resilience against shifting scenarios of social collapse. On this basis the paper problematises perspectives that either treat the social as an artefact of administrative practice or that prioritize experiences of moral purpose and commonality. It argues that such positions risk mythologizing ‘society’ if they don't attend to the complex circumstances of its political articulation.

## 1 Introduction

Perspectives on neoliberalism often take a strong, yet disparate, interest in the neoliberal impact on ‘society’. Some authors point to specific devastations that signal the neoliberal attack on ‘society’ (Fisher 2009, 19; Klein 2007, 109). Others refer to the ‘death of the social’ in the context of new paradigms of social administration (Dean 1999; Rose 1996). This ‘death’ can be seen to entail the end of future‐oriented deliberation on collective purposes that ‘society’, according to Giroux (2011), affords. Other authors invoke the loss of ‘society’ as they outline psychological deformations wrought by neoliberal rationality (Monbiot and Hutchison 2025, 60). From a different angle, contributions that trace neoliberalism's intellectual lineage point out its reliance on images of socio‐biological, ‘natural’ order (Davies and Gane 2021; Slobodian 2025). Neoliberals articulate reactionary ideas, often in direct opposition to progressive understandings of ‘society’, which neoliberal regimes proceed to enact with considerable coercive force (Bruff and Tansel 2020).

In extended reflections on neoliberalism, democracy and public life, Wendy Brown also centres ‘society’. For Brown, neoliberalism sets out to displace the social. Its success in this respect is seen in the systematic deterioration of democracy and public life where neoliberals hold sway. The impoverishment of collective imaginations about alternative futures, an ongoing process of meaning‐making that Brown associates with the term ‘society’, gives rise to ‘anti‐democratic culture’ (Brown 2019, 28): when ‘the social vanishes from our ideas, speech, and experience, it vanishes from our vision of the future, both utopian and dystopian’ (2019, 52). What follows is withdrawal from public life, the decline of democratic institutions, and the rise of morbid forms of ‘apocalyptic populism’ (Brown 2017). In an extended interview with Brown, Raza and Davison‐Vecchione (2024, 192) summarize this perspective as follows:

[By] negating the very notion of society or ‘the social’ as something experienced and tended in common, neoliberalism has ironically strengthened and hastened the nihilism of our age. This, coupled with neoliberalism’s accidental wounding of white male supremacy, has produced a kind of apocalyptic politics that would rather destroy the world than see a future in which this supremacy disappears.

Engaging with this position, I draw on Brown's perspective not to add to extensive debates on neoliberal techniques and rationality, but to reflect on how the concept of ‘society’ is mobilized, in this and other instances, and what critical engagement with its deployment requires. The argument in the following is that sociologists should attend to political agency that sketches out the social in response to changing historical circumstances, contextual requirements and political designs. Methodologically, this suggests an interest in the appearance of ‘society’ among disparate practices, experiences and institutions and as a reference point for policymaking, grassroots mobilisation and strategic politics. Following Lefort (1988, 18), my concern is to outline a position that ‘preserves [the] indeterminacy’ of the social. This perspective centres the political constitution of ‘society’ and its importance for imagining possibilities beyond neoliberal devastation, rather than the pursuit of social commonality that is always liable to being ‘lost’.

The paper makes this case conceptually, contrasting Brown's position with historical genealogies of the social (Castel 1995; Donzelot 1994; Ewald 2020; Procacci 1993), and empirically, by examining conspicuous articulations of ‘society’ in politics that pursues social cohesion. I consider the development, afterlife, and re‐emergence of social cohesion, tracking the articulation of British (*community cohesion*), French (*cohésion sociale*) and German agendas (*Bürgergesellschaft*) in the 1990s and 2000s, and their re‐articulation as vehicles for social resilience, domestic unity and strategic autonomy in the fraught context of the mid‐2020s (e.g., Macron 2024; MCHLG 2024; van der Leyen 2025). Working through this politics, my aim is to demonstrate the multiplicity of projects—top‐down, bottom‐up and in pursuit of utopia, solidarity, integration, regulation and domination—that invoke ‘society’. Social cohesion illustrates the versatility of ‘society’ and the coincidence of projects that, rather than offering a simple contrast with neoliberal rationality, set out to produce pro‐social subjects, invoke social togetherness or claim to defend ‘society’ against changing scenarios of social collapse. Attending to this versatility, I suggest that, much like broader claims about neoliberalism's demise, the neoliberal ‘death of society’ is a proposition in need of examination.

## 2 Society Must Be Defended

The understanding that neoliberalism harms ‘society’ appears, as noted, across a range of critiques of the neoliberal model. It often stands shorthand for more specific concerns, such as the dismantling of social protections, the privatization of collective goods or the decline of public institutions. Wendy Brown's elaboration stands out in that she appears to offer a more literal account of how ‘society’ is lost, accentuating functions and purposes she deems essential for the preservation of public institutions, for articulations of justice and for collective moral experience. The following unpacks Brown's argument and reflects on its significance, before considering her perspective among alternative accounts and origin stories of ‘society’.

Brown's work has been influential in shaping the debate on effects of neoliberalism across North American and European public life. In *American Nightmare,* she initially considered the ‘forces of de‐democratization produced at the intersection of neoliberal and neoconservative rationalities in the United States’ (Brown 2006, 691), setting the scene for a specific focus in *Undoing the Demos* (Brown 2015) and other work. *Nihilistic Times*, her most recent work, draws on Max Weber for a defence of ‘normative thinking’ against neoliberalism's ‘rampant nihilism’, whose effects need to be countered through value‐based, civic education (Brown 2023, 106–107, see also sect. 7, below).

Brown is not a sociologist, though her footprint in sociological literatures is substantial, and she appears prominently on outlines of foundational and advanced courses in political sociology and social theory as well as on the sociological conference circuit, such as in keynote presentations at the European Sociological Association conference. Beyond Brown's orientation as a critical‐social theorists, it is her conspicuous argument on the loss of ‘society’, and its defence as a site of meaning and commonality, that make her position relevant for this paper's broader concern with articulations of the social. Behind this discussion, there are questions about Brown's argument in relation to alternative critical‐sociological perspectives on the object of ‘society’ (Antonio 2024; Thorpe 2022) also in the context of crises and mobilizations to which (some) sociologists seek to respond (Burawoy 2022; Meghji 2024). There are questions, though they cannot be pursued here, about Brown's (2023) recent attempt to claim Max Weber for the purpose of post‐neoliberal restoration and how this should be read in the context of earlier debates about sociology's normative orientation (e.g., Gouldner 1961) and among the longer history of encounters between neoliberalism and sociological theory (Gane 2014).

To summarize, in *The Ruins of Neoliberalism* (2019) Brown places ‘society’ at the centre of her argument. As noted, the rejection of social concern and the denunciation of political projects that conceive of social objectives is distinctive for advanced neoliberalism. The disappearance of the social is felt most sharply in the absence of shared understandings and common political imaginaries. The ‘disintegration of society and the discrediting of the public good by neoliberal reason’ (Brown 2019, 7) make resources of commonality precarious. Recurrent ‘attacks on society understood as experienced and tended in common’ (Brown 2019, 15) prepare the ground for powerful forms of nihilistic, anti‐democratic politics. The demonization of the social creates the space for far‐right movements to attack ‘social justice warriors’ and repudiate movements for racial equality. It underpins state interventions that no longer merely speak, but conduct policy and govern, in increasingly authoritarian and fascist terms (Brown 2017). Unshackled from ‘society’, reactionary forces draw energy from the denunciation of progressive politics and the hyper‐individualist celebration of raw power.

The timeline Brown suggests for the loss of ‘society’ tracks the programmatic articulation of neoliberal ideas and their rise to dominance in political‐economic governance. Her argument clearly prioritizes, though it extends beyond, cultural and political‐economic change in the United States, where claims for social justice become the subject of angry denunciations in the name of individual freedom and ‘traditional values’ (see also Brown 2023, 2017). The fragmentation of American cultural and political life exemplifies what happens when the social has been lost. The destruction unleashed by the second Trump administration, and by newly empowered far‐right governments elsewhere, needs to be seen in this post‐social context.

Brown's request for the recovery of ‘society’ is not ‐ at least not explicitly ‐ for the return to social democracy, for the security the post‐war welfare state or for the social re‐embedding and democratic re‐encasing of markets. Rather, by conceiving of society as the ‘place where we experience a linked fate across our differences and separateness’, Brown (2019, 27) sidesteps such functions of the social in favour of collective meaning‐making and for the safeguarding of public institutions that democracy requires. It is liberal arts education and Brown’s (2015, Ch. 6; 2023) own pedagogic practice that exemplify social potentials against the onslaught of ‘nihilistic’ politics.

The aim in what follows is to consider the role that ‘society’ plays in this account, not if Brown succeeds in offering a convincing perspective, for example, on the contemporary resurgence of the far‐right and its ‘culture wars’. Equally, it is not my goal to compare her work to richer perspectives on neoliberalism's intellectual and institutional origins (e.g., Slobodian 2018; Stedman Jones 2012). These show that, as a matter of intellectual history and strategic alignment, neoliberalism's affinity with far‐right politics is more than a contingent encounter and closer than Brown suggests (e.g., Bruff and Tansel 2020). Slobodian (2025, 15) highlights the appeal to biology, and its ‘fusion’ with economic doctrine, as neoliberals strategize the ‘painful transition out of the world of the social state’ and join up with far‐right, neo‐fascist politics. At times, this alignment occurs not against, but in the name of ‘society’, understood as an artefact of socio‐biological evolution and defined by the commonality of ethno‐racial or gendered ‘difference’ (see also, e.g., Sharpe 2025).

Brown does not attend to such complexities and offers only a short summary account of neoliberal political‐economic paradigms, drawing on Hayek's and Friedman's writings. Based on this, she develops the central claim of her 2019 book, which is this paper's primary focus. Brown (2019, 28) argues that

the existence of society and the idea of the social—its intelligibility, its harboring of stratifying powers, and above all, its appropriateness as a site of justice and the commonweal—is precisely what neoliberalism set out to destroy, conceptually, normatively, and practically

(Brown 2019, 28)

As noted, the disappearance of society forecloses the deliberation on collective futures because ‘the language of the social is where subjections, abjections, and exclusions are lived, identified, protested and potentially rectified’ (Brown 2019, 40). It shuts down the articulation of a sense of collective hope and of a shared ‘vision of the future, both utopian and dystopian’ (Brown 2019, 52). As the social is ‘the essential modern site of emancipation, justice, and democracy’ (Brown 2019, 50), its disappearance removes an indispensable anchoring point for collective political experience and democratic practice.

Brown appears to conceive of the social as a space of unconstrained deliberation about collective solidarity and desirable futures. She seems to treat the relative presence or absence of ‘society’ in political language as determinative for the quality of political projects that speak in such terms as well as for the quality of public communication and democratic exchange. Arguably, this is not for the substantive outcomes (such as ‘justice’) or procedural commitments (such as ‘inclusion’) that politics in the name of ‘society’ envisages, but for certain imaginary resources ‘society’ makes available. For Brown, the social matters for one reason: it provides resources of commonality that take us beyond narcissistic individualism and nihilistic relativism (Brown 2019, Ch. 5; see also 2023).

Elements of this account seem intuitive and align with other, widely‐cited discussions of neoliberalism's social impact (e.g., recently, Monbiot and Hutchison 2025). Neoliberalism, as a political project, sets out to depoliticize economic governance and, as Slobodian (2018) puts it, ‘encase’ markets from democracy, removing them from the sort of collective meaning‐making that Brown envisages. Empirically, her perspective aligns with the increasingly open dismissal of popular democracy as a desirable mode of government by right‐wing libertarians in the United States. The relevant anti‐democratic tenet, also reflected in the mindset of Silicon Valley technofeudalists like Thiel (2009), is that ‘the believer in freedom has never counted noses’ (Friedman [1992] cited in Slobodian 2023, 32).

Brown's perspective on the social as the indispensable site for commonality burdens ‘society’ with considerable weight. As I will argue below, it fails to account for the complexity of society's political articulations. What disappears, moreover, is what Donzelot (1991, 175) calls the ‘statistical nature’ of the social. Political deliberations about ‘society’ may point towards collective futures, but they equally proceed with an interest in regularities and averages, through the paternalistic determination of ‘needs’, and are expressed in a concern for stability, order and integration. The constitutive concern with ‘society’ tracks such complex considerations much more than Brown's emphatic concern with democratic resources. So do, to a significant extent, available reflections on the disciplinary origins of sociology.

Quetelet ([1869] cited in Ewald 2020, 79) articulates the statistical nature of the social as the requirement that

we must abandon the view of man taken in isolation and only consider him as a part of the species. By stripping him of his individuality, we will eliminate all that is accidental; and the individual particularities that have little or no effect on the masses will disappear of their own accord and allow us to grasp the general results.

This perspective is captured most systematically by French historians of the ‘problem of the social’ as they build on Foucault's (2003, 2004) concern with ‘the population’, engaging with the institutions, practices and forms of knowledge that underpinned the emergent welfare state and the social vocations that Castel (1995) refers to as ‘faire du social’. The following contrasts this administrative perspective on society with Brown's defence of ‘society’ as a container of social commonality and moral meaning. First, it probes the developmental logic underpinning claims about the loss of ‘society’.

## 3 Nostalgic for What?

Brown's comments regarding the displacement of society form part of a broader argument regarding the disappearance of democracy and the decline of public institution. It is important to note the nostalgia attached to problematic antecedents that this account of subtraction and decline requires. It is one thing to concede, as Brown (2015) does, the injustices that preceded ‘neoliberalism's stealth revolution’. But this acknowledgement is no substitute for some engagement with the conditions of possibility for the civic democracy and for the actualized social that neoliberalism is said to have destroyed. The revitalization of public institutions and the recovery of collective political imaginaries provide the antidote to rampant ‘nihilism’ (Brown 2019, Ch. 5; 2023). This prescription isn't burdened by suspicion that dominations and exclusions, for example those that facilitated the pre‐neoliberal ‘golden age for public higher education’ (Brown 2015, 180, see also sect. 7), may have been constitutive for what has been lost.

The broader point is that the commonality that Brown requires does not present itself for unproblematic re‐discovery (this is an insight that Young 1990, in particular, insists upon). Even if the social is conceived as a domain of meaning‐making (and we sideline the space of social regulation, see below), aspirations towards commonality will be the subject of competing claims and assertions of difference based on distinctive ‘ideals of liberation’ (Young 1990, 163) that may not be irreconcilable, but certainly will be contested.

Without much interest in its conditions of possibility, Brown is only able to offer a streamlined, developmentalist account of the social and a nostalgic vision of its restoration. At its most basic, this is the idea that neoliberalism subverted a space of inclusive solidarity and democratic togetherness that had been in place up to the 1980s, when the neoliberal intrusion into social, political, economic and cultural spheres began. This developmental account, conceiving of neoliberalism as destructive of inclusive solidarities that been enshrined in the post‐war welfare states of Europe and North America appear, *should* appear questionable. It only works with reference to powerful myths, be it about the *Great Society*, the *trente glorieuses*, the *Wirtschaftswunder* or the heroic discovery of post‐war welfare democracy. It elides the relationships of exclusion and extraction that, it has been convincingly argued (Bhambra 2022; Boatcă 2016; Lessenich 2016), constituted such models of solidarity and continue to secure what remains of them.

Rather than relying on a nostalgic account of the social that is thought to have preceded neoliberalism, the urgent task that others identify is to reconstruct solidarity in a way that doesn't reproduce relationships of subordination and extraction (Bhambra and Holmwood 2018, 584; Lessenich 2020, 127–128). This is not a purely theoretical challenge. It is instantiated by powerful projects with pro‐social claims, harking back to mythical ideas of solidarity, including those that centre on the ‘white working class’ (Shilliam 2018; Dobbernack 2024). The radicalization of exclusionary practices among Nordic social democracies, generally justified in pro‐social terms, gives further cause for concern about the terms of any social restoration. Claims about the ‘death of the social’, and the request for its re‐discovery, need to attend to the exclusions, material preconditions and global relations that sustain the presence of ‘society’.

## 4 Inventing Society

Without much acknowledgement, Brown's ideas are reminiscent of earlier perspectives on the disappearance of ‘society’ by scholars of governmentality who grappled with the prominence of ‘community’. Authors commented on the direction of social policy they witnessed, extrapolating from political language that invoked society, such as Thatcher's rhetoric (‘there is no such thing’) or the emergence of new political slogans around conceptions of community, culture or citizenship. What distinguishes their position from Brown's is that they offer a more complicated genealogy of ‘society’ and its content. Although Rose (1996, 2000), for example, certainly overstates the emergence of ‘community’ in the 1990s, for him the social is not a fixed domain laden with pre‐defined political purpose. It emerges through processes of ‘discovery and re‐discovery’ (Dean 1999, 54) and in a context of mobilizations but also of changing bureaucratic, scientific and regulatory practice. This perspective brings into view not just the diminishment of social purposes in neoliberalism or the sloganeering of 1990s neo‐social democrats (New Democrats, New Labour, *Neue Mitte*). It allows for a critical view on the definition of social needs, on practices of social regulation and on the objective to maintain social unity and stabilize social order, often with an emphasis on the pacification of working class, all in the name of the social.

Claims about the vanishing of the social need to be seen, then, in the context of society's continued discovery and ongoing re‐invention, in relation to regulatory projects and as the subject of emancipatory politics that challenges the ‘statistical nature of the social’ (Donzelot 1991, 175). Deleuze (1980, ix) sees the beginning of this discovery ‘in the late eighteenth and early nineteenth centuries, [when the social] sketches out its own originality in relation to older sectors and is able to react on them and effect a new distribution of their functions.’ Historians of social administration, such as Castel, Donzelot and Ewald, locate this moment among emergent social problems requiring new regulatory practices. Donzelot (1994, 18) situates the ‘invention of society’ at the point when ideals of freedom and equality—and an expanded franchise—encountered the material reality of industrial society, offering a perspective that draws in social administration and political mobilization. Castel (1995) suggests that the resulting ‘social question’ emerged around the condition of impoverished populations and how they ought to be accommodated, managed, improved and included. Ewald (2020, 77), as mentioned above, draws on Quetelet to trace the ‘statistico‐probabilistic objectification of society’. Ewald's intellectual trajectory, from Foucault's doctoral supervisee to figurehead of French employers' association, is complex; it is his perspective on practices of social regulation that is of interest here, which Ewald no longer saw as repressive but as ‘open and playable’ (Ewald cited in Behrent 2010, 587).

Following a different intellectual lineage, Boltanski and Chiapello (1999) capture the multiplicity of normative expectations and spatial understandings entailed in images of ‘society’ that co‐exist. Among their most influential claims is that newly dominant images ‘society’ make the capacity for activity, through the flexible pursuit of projects and networking, important criteria in the determination of human worth. In comments on welfare restructuring that tracks this work, Lessenich (2008, 76) suggests that through the (related) ‘reinvention of the social’ the redistributive state is replaced by the *Aktivgesellschaft*, a social world that channels the subjectivity of citizens towards regulatory purposes which are presented as consistent with the ‘spirit of the social’ (2008, 122).

Such perspectives on regulation and governance, and the perspective on ‘society’ as an artefact of administration, don't have to discount the social as a space for collective deliberation and articulations of justice. But they suggest that ‘society’ cannot be reduced to such imaginary investments. The social presents itself for a multiplicity of claims, practices and projects of meaning‐making and regulation. What is missing from perspectives that narrowly focus on either regulation or commonality is the role of social‐political articulations that make ‘society’ visible in the first place.

## 5 Beyond Regulation and Commonality

To summarize, it is possible to schematically distinguish two understandings of ‘society’, though (as I will argue below) any such division that remains blind to its counterpart runs into trouble.

1. Brown's ‘society’ appears as a space of symbolic investment and utopian imagining. Her account resembles theorizations of the public sphere that single out resources of collective meaning, often presented as fragile, yet essential for democratic communication (e.g., Habermas 1989). It is an account of the social as an emphatic space or moral purpose and social unity, which prioritizes democratic communication and shared political and cultural experience.
2. Historical sociologists of ‘society’, taking cues from Foucault, trace the emergence of society with emphasis on knowledge, institutions and regulatory practice. The social is conceived with reference to administration and bureaucracy responding to social problems and social needs as much as defining what such problems and needs are. This often presents itself as an account of the statistical nature of the social, which tracks imperatives of social integration and the administration of society in pursuit of historically contingent, regulatory objectives.

In this form, this is a blunt distinction, and different modalities of the social have been outlined with considerably more nuance (from different angles, e.g., by Boltanski and Thévenot 1991; Donzelot 1991). Yet separated in this form, it becomes possible to ask how claims regarding the neoliberal attack on the social, and its alleged vanishing, could be conceived or articulated in either modality as well as what both may be missing.

In the first, and in alignment with Brown, this might be neoliberalism's interference in collective deliberations and its individualizing thrust. It could be the anomia that results from the destruction of collective meaning; or it may be the spread of fatalistic perspectives on social circumstances as unchangeable and beyond human control. In the second, this might be the loss of the social state—understood as the state that ‘does the social’ (Castel 1995)—and the emergence of regulatory techniques that sidestep the domain of ‘society’, for example the ‘etho‐political’ projects Rose (1999) identified; it could be the emergence of new sites, beyond the territorial state, and subjects, beyond the population; it may include the arrival of new political strategies to ‘encase’ markets and diminish social security.

Yet any non‐reductive attempt to determine neoliberalism's relationship with ‘society’ will struggle to conceive of these two modalities in isolation. Regulation and meaning‐making need to be considered for how they align as this how ‘society’, beyond any schematic conception that relies on pre‐determined meaning, presents itself whenever it becomes concrete and specific. This is exemplified, as I will argue below, by social policy agendas that aim for regulatory intervention and social governance while invoking emphatic ideas of social unity and togetherness. But it is equally conspicuous in bottom‐up claims about the social that, as soon as they offer more than minimal determinations of what equality and justice require, give at least a partial account of the population, its features, composition and the needs that require satisfaction as well as deciding the purposes of the state that is charged, following Castel (1995), with ‘faire du social’.

To be clear, it is not *just* the elevation of commonality in Brown's writings on the social that is the issue, then, but also its mirror image among scholars of social administration. Work that treats ‘society’ purely as artefact of regulatory and administrative practice, without accounting for potentials towards its political imagination and democratic constitution, also falls into reductionist traps. This is a matter of degree among Foucault‐inspired perspectives that grapple with the ‘government of the social’. At its most extreme, François Ewald shrinks the space of politics to a minimum when he suggests that political debate ‘over the crisis of the welfare state [implies nothing but the determination of] the best modalities of managing it’ (Ewald cited in Behrent 2010, 612).

Treated in isolation, what is missing here is not just the link between two conceptions of ‘society’. It is a measure of interest in the exercise of social imagination as well as in the operation of political language that make the social visible in the first place. The most common understandings of society that are in circulation, of course, demonstrate this in their reliance on metaphorical representation. The social, as Wrong (1994, 4) suggests, is ‘redolent of physical, mechanical, chemical, and biological imagery’. Arguably, claims about the presence or absence of ‘society’, its decline and disappearance, or the emergence of new metaphors of togetherness, reflect changing metaphorical accounts. Such changes may well demonstrate the neoliberal attack on ‘society’, in so far as prevailing political‐economic circumstances render certain understandings less plausible, intelligible or resonant, or signal a more general departure from ‘social talk’. Yet they may equally demonstrate that ‘society’ appears, and has the potential to re‐appear where it is thought to have vanished, in fleeting, complex and contradictory ways.

My argument, then, is that articulations of ‘society’ matter. It is an important objective for political sociologists to consider claims about ‘society’ and to attend to different forms of political agency that sketch out the social. The following section traces a conspicuous example for such political articulations by considering the European politics of social cohesion. It comments on the versatility of ‘society’ as political reference point and notes the coincidence of commonality and regulation in three contextual snapshots.

## 6 The Social in Social Cohesion

Claims about the vanishing of the ‘society’ contrast with the prominence of social themes and priorities in European social policy. The social is frequently articulated with reference to states of integration, order and unity, and expressed in terms such as *gesellschaftlicher Zusammenhalt*, *community cohesion* or *cohésion sociale*. Social cohesion is not the only social priority that would illustrate the importance of such articulations in determining what ‘society’ entails. Yet, as the headline title for changing policy agendas, it illustrates contextual differences and the wide scope actors have as they invoke the term for different social‐political purposes.

In the 1990s and 2000s, policy makers often referenced cohesion in relation to desirable forms of pro‐social conduct, singling out attitudes among ethnic‐minority populations (UK) or welfare recipients (France). More recent articulations take a new, defensive tone. In the 2020s, European political leaders draw attention to newly powerful strategies that seek to heighten tension, provoke crisis and dismantle liberal‐democratic unity. Cohesion becomes the all‐purpose remedy for social resilience and, in some cases, against the divisive impact of the far right. This new, defensive posture is more than mere metaphor: EU member states have now been invited to decide ‘if they want to use cohesion policy programmes, to increase defence spending’ (van der Leyen 2025).

There is variance, then, in how cohesion is articulated and how it appears and disappears, is invoked or rejected in the pursuit of political projects, including within political spaces that have been profoundly shaped by neoliberal governance. Depending on how cohesion is filled with meaning, it evokes aspirations of future social togetherness and channels ideas of commonality; at least this is characteristic for much of its political rhetoric. Yet the contrast between states of social harmony and dystopian scenarios of disintegration and collapse doesn't just play out in a symbolic or rhetorical space. In claims about cohesion, the social *also* instantiates political‐strategic positioning and a battery of regulatory interventions that, proponents of cohesion argue, are needed to achieve desired social futures, avert catastrophic social collapse, govern ethno‐religious pluralism, establish popular buy‐in for redistributive measures, and—more recently—create a bulwark against the resurgent far‐right.

### 6.1 Cohésion Sociale

Addressing the frames of French social policy, Donzelot (2006, 3) suggested that, through the 1990s and into the early 2000s, *cohésion sociale* had come to take the place of *progrès sociale*. The republican state ceased to present itself as ‘the guarantor or custodian of social progress’ and began to conceive its function as to incite ‘civil society to produce cohesion in a competitive environment’ (Donzelot 2006, 11), a suggestion that tracks with the turn towards ‘activity’ and the flexible pursuit of projects captured by Boltanski and Chiapello (1999).

As a political priority, the conspicuous turn towards cohesion followed Chirac’s (1994) socio‐moral invocation of *fracture sociale*. The subsequent discovery of *cohésion sociale* occurred in the text of far‐ranging welfare reform agendas into which Chirac's administrations invested considerable effort, before pulling back (in, by now, familiar patterns) in the face of social protest. A *loi d'orientation relatif au renforcement de la cohésion sociale* was meant to be passed in April 1997 but failed when Chirac, in a gamble, dissolved the National Assembly and lost the following parliamentary election. The 2004 *plan de cohésion sociale* (named after the minister Jean‐Louis Borloo), the culmination of reformist zeal during Chirac's second presidency, a broad programme of measures was eventually reduced to a limited programme of upskilling in the labour market.

With Chirac's departure, *cohésion sociale* ceased to be used as a frame for reformist policy until, in his New Year's address, Macron (2017) declared 2018 to be the ‘year of national cohesion’. Instead, 2018 saw the emergence of widespread protest in the form of the *gilets jaunes*. Yet social‐integrationist language continues to play an important role in recent policy initiatives. Regarding pensions reform, Macron (2023) acknowledged divisions and anger, which he traced to pervasive sense of decline and deterioration (*déclassement*) and the feeling of abandonment. The cause that Macron invokes for this purpose is distinguished by consistent emphasis on France's sovereign independence:

We are a people who intend to control and choose their destiny. We do not want to depend on anyone: neither on the forces of speculation, nor on foreign powers, nor on other wills than our own.

(Macron 2023)

Welfare restructuring is presented as prerequisite for the maintenance of sovereign independence, which Macron frames (in contrast to the appeal to sovereignty by far‐right rivals) as an objective to be pursued in a European frame.

Analogous to Chirac's reference to *fracture* and *cohésion sociale*, Macron's (2023) language is redolent with metaphors of movement: ‘rebuilding and regaining the momentum for our nation’; achieving ‘national momentum’; ‘releasing energies’; creating ‘more freedom of action, experimentation, power of initiative’. The idea of social unity, articulated in the face of substantial protest and widespread discontent, continues to require collective mobility and individual activity: ‘*la cohésion de la Nation,* […] c*'est le travail de chacune et chacun d'entre vous*’ (Macron 2017).

The requirement for everyone to ‘pull together’ draws on continuous references to utopian potentials, expressed in the emphatic language of republican commonality. In programmatic speeches on pension reform, Macron (2023) invoked the decision to rebuild Notre Dame after its partial destruction. Cynics, he argued, had deemed this project to be impossible, but it would be achieved, thanks to Macron's spirit of initiative and through the ‘mobilization of everyone’. The ‘nation's major construction sites’—in particular, the reform of allegedly unsustainable systems of collective security—needed to be addressed in the same way. In a seamless shift from such reformist objectives, Macron (2024) invokes *cohésion sociale* for Europe's distinctiveness, underpinned by forms of social solidarity that he presents as specifically European and that lend support to his renewed call for strategic autonomy from the United States.

### 6.2 Community Cohesion

Before Labour's landslide victory in 1997, Blair cast the circumstances of Thatcherite politics as a moral crisis, presenting the moral disorientation caused by Conservative governance as root cause of major social problems. Blair (2010, 57) himself noted how ‘effectively’ he had managed to turn the highly mediatized murder of the toddler James Bulger ‘into a symbol of a Tory Britain in which, for all the efficiency that Thatcherism had achieved, the bonds of social and community well‐being has been loosed, dangerously so’. Labour entered government committed to this socio‐moral agenda, which was beginning to be captured in the commitment to ‘cohesion’, a term that, though couched in communitarianism, would be employed to prioritize behavioural solutions to social problems. Anti‐social behaviour orders (ASBOS), a set of penalties and restrictions imposed on people accused or suspected of anti‐social conduct, exemplified the punitive edge of Labour's pro‐social agenda (Squires 2008).

Labour's new socio‐moral orientation was deployed in official accounts of urban unrest in Northern towns in 2001, when it found an expression in the concept of ‘community cohesion’. In the development of this frame by Cantle (2001) and others, ‘community cohesion’ highlighted in particular the consequences of ‘self‐segregation’—the idea that ethnic communities were inward‐looking and parochial. The breakdown of Britain along ethnic lines, the ‘sleepwalking’ into segregation that Trevor Phillips invoked in his account of ‘nightmare’ of ‘fully fledged ghettos’ (Phillips 2005), or of Bradford in ‘the grip of fear’ (Ouseley 2001, 1), facilitated the turn towards community cohesion as an integrationist remedy. It allowed for a reading of urban unrest, among a host of other social problems in Britain's multicultural spaces, as indicative of a social malaise that required new dispositions, attitudes and values among minority populations. Their behavioural problems were presented as the result of a laissez‐faire multiculturalism and the failure to insist on shared values—a causal story that remained popular after Labour's exit from power (Cameron 2011).

In the post‐Brexit context, Boris Johnson proposed a focus on regional inequalities and on ‘left behind’ places and populations. Even as a rhetorical device, the emphasis on ‘levelling up’ has turned out to be less impactful than anticipated by political observers (e.g., Jennings et al. 2021). From the centre left, the new Labour government invokes patriotism and service of ‘working people’, without relying on an emphatic conception of the social. There are signs that ‘cohesion’ is re‐emerging as a political priority (MCHLG 2024), also in view of racist riots that reverberated through the country since the summer of 2024, though its meaning is yet to be determined.

### 6.3 Bürgergesellschaft

The lineage of integrationist language in Germany is complex (see, e.g., Conze 2004); in the late 1990s and 2000s, public intellectuals and government authorities began to rely on the notion of *Zivilgesellschaft* and *Bürgergesellschaft* to articulate a reformist vision of the social. Staking out a claim to conceptual novelty, Schröder (2000) invoked *Zivile Bürgergesellschaft* in his government's push towards sweeping welfare and labour market reforms of the *Agenda 2010.* In addition to its role a blueprint for democratic reform, *Bürgergesellschaft* promised to remedy a crisis of comparative competitiveness, traced to the inertia of the German socio‐economic model, framed as a new turn towards individual self‐reliance and collective mobility.

Following the reformist initiatives of the Schröder government, there was a relative absence of socio‐moral jargon in response to perceived crises, and fewer top‐down political projects that would speak on behalf of ‘society’ either in terms of cohesion or for its desired mobilizations. The Merkel era entailed a degree of satisfaction with the country's macroeconomic outlook, but also by substantial challenges—not least the *Flüchtlingskrise*—that may have been open to, but didn't receive, a coherent social framing. Only more recently has ‘social cohesion’ re‐emerged as a focus of heightened programmatic concern.

The emphasis on *Zusammenhalt* is now pervasive but largely unspecified. In the coalition agreement between Conservatives and Social Democrats (Koalitionsvertrag CDU/CSU and SPD 2018), the term made a headline appearance, yet remained substantively vague, invoked as self‐explanatory objective in relation to ‘cultural diversity’, ‘cultural politics’ and ‘democratic renewal’ (Ch. XIII). In 2025, a new agreement between the same parties prioritizes *Zusammenhalt* against ‘hybrid attacks on our country that aim to destroy cohesion, undermine democracy and endanger our security’ (Koalitionsvertrag CDU/CSU and SPD 2025, 1). Among the many, ambivalent objectives cohesion supports is a new crackdown on undesired immigrants and new deportation measures (2025, 94).

The Federal Government has made a substantial investment in research on the circumstances of cohesion, establishing a *Forschungsinstitut für gesellschaftlichen Zusammenhalt*, with representation at 11 German universities and 40 million Euro funding. Social scientists in charge of this initiative emphasize the intrinsic openness of the *Zusammenhalt* (Deitelhoff et al. 2020; Forst 2020), which is mirrored in political rhetoric where *Zusammenhalt* permits contradictory objectives. Cost of living pressures (e.g., RND 2022) are as much open to this framing as arguments about the harmful fragmentation resulting from ‘identity politics’. Regarding the latter, the left‐wing nationalist Sahra Wagenknecht, who had become widely visible for attacks on the government's Covid‐19 measures, as critic of its support for Ukraine and, since 2024, as the leader of an eponymous party, centrally invokes *Zusammenhalt* in her claim to speak on behalf of ‘ordinary’ (ethnic) Germans and to bridge the divide between left and far‐right politics (Wagenknecht 2022). Others invoke *Zusammenhalt* against the fragmentation and divisiveness they associate with Wagenknecht and Germany's resurgent far‐right.

## 7 Demythologizing Society

Before considering the politics of social cohesion further, let us return to Brown's account of ‘society’. Brown (2019, 46–50) juxtaposes her perspective with Arendt’s (1958) dismissal of ‘society’ as the domain of dehumanizing conformity. It is interesting to note how Hanna Fenichel Pitkin, Brown's one‐time Berkeley colleague, works through this intended meaning as she offers the most extended treatment of Arendt's perspective on the social in *The Attack of the Blob* (Pitkin 1998). Pitkin suggests that the social, for Arendt, closely corresponds with what ‘alienation’ meant for Marx: loss of agency and control, with corresponding damage to personal dignity and self‐regard. Human collectives find themselves operating on ‘social’ terms when they ‘conduct themselves in such a way that they cannot control or even intentionally influence the large‐scale consequences of their activities’ (Pitkin 1998, 16, see also 177). Pitkin (1998, 251–252) sets out to ‘demythologize’ Arendt's perspective on ‘society’ and suggests an alternative view on the social as ‘complex composite’ (1998, 17). It is not possible to pursue this line further, except to suggest that Brown's account arguably re‐mythologizes ‘society’. Different from Arendt, it now becomes associated with the sole purpose of meaning‐making for the production of commonality and to fortify a preferred zone of civic and educational practice. The paradox in this is that ‘society’, for Brown, is essential for progressive politics and democratic communication. Yet it is removed, if not fully separate, from democratic contestations and constitutive claims that would give ‘society’ meaning. As noted above, though post‐Foucauldian approaches sometimes bemoan the ‘mystification of the social’ (Deleuze 1980, x), they often betray a similar lack of interest in social‐political agency that, depending on one's preferred perspective, constitutes ‘society’ or has at least a role to play in making ‘society’ visible.

The three agendas considered above provide tenuous case material that speaks, more than anything, to the elasticity of political language. Yet this is their point. Real‐world articulations invariably demonstrate how ‘society’, and the appeal to resources of social commonality, appear in fleeting, contradictory and complex ways. The social can become the site for claims about equality and justice and for open‐ended deliberations on collective futures that, according to Brown, democracy requires. But references to ‘society’ equally permit the articulation of regulatory objectives and the pursuit of stabilizing interventions, often in pursuit of unity, order and societal integration. Attending to this complexity, it is not clear that neoliberalism—understood as the prioritization of markets, the dismantling of collective security or the adoption of governing techniques that aim at individual behaviour—requires the ‘death of the social’. It isn't evident that neoliberal politics militates against the presence of ‘society’ as a frame of reference, especially not if ‘traditional values’ or biological sameness are in the centre of this social frame. Moreover, it is a shared tendency in the politics of cohesion that emphatic references to ‘society’ underpinned the push towards welfare restructuring and policies of activation, aiming at groups and individuals portrayed as insufficiently attuned to desirable social purposes, cast as harmfully anti‐social and out of line with the required spirit of commonality. Despite its reliance on pro‐social rhetoric and utopian framings, cohesion had role to play in the broader neoliberal project. Though the three snapshots presented above don't fully substantiate this claim, I argue that it set politics on an accelerated path towards activation, conditionality and behavioural governance (Dobbernack 2014).

The complex appearance of ‘society’ requires more careful consideration, then, for the role that the emphatic appeal to commonality plays in projects of behavioural regulation and social governance. Other suggestions are available, for example by working through the complicated legacy of authors who considered the ‘government of the social’ (Castel 1995; Donzelot 1994; Ewald 2020). In work on ‘the post‐soviet social’, Collier (2017, 12) examines how ‘elements of social government and neoliberalism appear together’ and suggests that the ‘death of the social’ fails to notice contingent assemblages among advanced liberalism and the social state. Elliott and Turner (2015, 824) point towards ‘creative everyday processes of sociality’ that, they argue, defy claims about the death of ‘society’, although I am not certain about the relationship between such processes, administrative practice and political language in their work. The three agendas of cohesion, presented as mere snapshots, reinforce this view. They illustrate that conspicuous emphases on social commonality coincide with a variety of political ends and administrative designs.

This would seem to complicate Brown's account along with other perspectives claiming the ‘death of society’. Yet there is a further point to make regarding Brown's social agenda, which regards the sites, institutions and experiences that she privileges as she outlines the potentials of ‘society’. Despite many ‘ghastly episodes and wrong turns’, Brown (2015, 180) considers the North American 20^th^ century as the ‘golden age for public higher education’, providing a template for the achievement of social potentials through liberal arts education. Engaging with Weber, Brown (2023, 92) articulates this ideal, and that which has been lost, as the production of ‘an informed, politically engaged citizenry [whose development is] an academic mission’. Today, the objective for liberal arts education is to ‘right our own ship’ (2023, 97) and to ‘preserve the scholarly realm’ (2023, 98). This means combatting the intrusion of market logics into university life. It also means, as Brown (2023, 106) asserts with some ambivalence, ‘dismantl [ing] the shrill and relatively anti‐intellectual character of the politicized academy today, offering in its place more productive, and more intellectual, practices of relating academic and political life’. The objective is to consolidate higher education against ‘nihilism’ from across the political spectrum. It is to return to the high‐water mark of public mindedness at work in American liberal arts education of the pre‐neoliberal 20th century. Conceiving of this restorative vision with a measure of cynicism, it appears Brown (1995) exhibits a form of ‘wounded attachment’ to the lost potentials of her own professional niche.

## 8 Conclusion

My main aim has not been to offer an unforgiving reading of Brown, who acknowledges that her approach is eclectic (e.g., Raza and Davison‐Vecchione 2024, 194). Neither is it to take issue with the centrality Brown attributes to ‘society’ in her account of neoliberalism, which mirrors social devastations that have been widely noted. Rather, it is to complicate what ‘society’ means and argue for more sustained interest in its political articulation. This is a necessary complement, I suggest, for any perspective that approaches the social either solely as a site of commonality and moral meaning or conceives of it purely as an artefact of administrative practice.

Beyond its detachment from democratic‐constitutive practice, Brown's account of ‘society’ presents further issues. It draws on problematic antecedents and is construed so that it always appears at risk of being lost if not for the pedagogic practice of self‐appointed guardians. This rarified, artificially stabilized conception of ‘society’ cannot be anything but nostalgic. It is of questionable value against the apocalyptic politics that Brown, in contrast to her earlier attempts to engage with the far‐right by grappling with fantasy and desire, now seems to trace predominantly to the loss of moral meaning.

More generally, my argument is that that we need to acknowledge the multiplicity of political projects that ‘society’ affords. These are varied and internally complex. They may, but don't have to, invoke horizons of commonality, some of which draw on progressive objectives whereas others posit solidarities in the name of ethno‐national groupness or ‘traditional values’. From a different angle, social movements regularly demand institutional solutions to social problems without necessarily appealing to strong versions of social commonality. Conversely, public administrators and social policy‐makers frequently present their objectives with reference to an emphatic, utopian social. The presence of ‘society’ in such instances does not tell us much about the quality of democracy, nor does its absence signal its neoliberal death.

One of the tasks for political sociology, then, is to interrogate articulations of ‘society’ in bottom‐up and top‐down politics, rather than taking its meaning for granted based on preferred conceptual schemes. This work is likely to reveal, as Dean (2010, 681) notes, that ‘the idea of ‘society’ is not an emancipatory domain confronting a dominating state’. Likewise, it is bound to show that the social is not well understood if it is conceived as moral resource that sets societies on a progressive and democratic course.

The social in social cohesion illustrates this political openness. The programmes outlined in sect. 6 channelled ideas of social togetherness in pursuit of French welfare reform or towards the post‐multiculturalist transformation of UK policy practice. Agendas of social cohesion emerged in competitive environments, reflecting the ambition to frame social‐moral challenges and ‘own’ relevant conceptual languages. Scenarios of disintegration, fragmentation and polarization—the ubiquitous German emphasis on *Spaltung der Gesellschaft*—can be counterposed with images of social unity and commonality. Yet this contrast does not simply fuel democratic practice nor does it invariably make space for collective ventures and shared experience. It underpins variable politics that, in different circumstances, aims for emancipatory and reactionary objectives, pursued through individualizing techniques of behavioural governance and other measures that don't fit the neoliberal bill.

It is a problematic move, then, to position ‘society’ in strict opposition to neoliberalism, especially not if this framing relies on nostalgic periodizations of social‐democratic public‐mindedness, irrevocably destroyed by the intrusion of neoliberal governing rationality. At least in Europe, ‘society’ has not disappeared as a reference point, although agendas of social cohesion have certainly ebbed and flowed. There is little reason to think that new governing programmes, as well as movements from below, will not re‐claim ‘society’ in politically suitable ways. Yet this doesn't tell us much about the fate of neoliberal governance, including fiscal austerity, the transfer of public goods into private ownership, the dismantling of social protections, policies of activation or regimes of welfare conditionality. After all, the former British Chancellor of the Exchequer, Osborne (2011) claimed ‘we are all in this together’ and invoked a ‘strong society’ as he set the UK on its lasting track towards austerity.

## Conflicts of Interest

The author declares no conflicts of interest.

## Data Availability

The author has nothing to report.
